# Grains, components and mixtures in biomedical ontologies

**DOI:** 10.1186/2041-1480-2-S4-S2

**Published:** 2011-08-09

**Authors:** Ludger Jansen, Stefan Schulz

**Affiliations:** 1 Institute of Philosophy, University of Rostock, 18051 Rostock, Germany; 2 Institute for Medical Informatics, Statistics and Documentation, Medical University of Graz, Auenbruggerplatz 2, 8036 Graz, Austria

## Abstract

**Background:**

In biomedical ontologies, mereological relations have always been subject to special interest due to their high relevance in structural descriptions of anatomical entities, cells, and biomolecules. This paper investigates two important subrelations of **has_proper_part**, *viz.* the relation **has_grain**, which relates a collective entity to its multiply occurring uniform parts (e.g., water molecules in a portion of water), and the relation **has_component**, which relates a compound to its constituents (e.g., molecules to the atoms they consist of).

**Method:**

We distinguish between four kinds of complex entities and characterize them in first order logic. We then discuss whether similar characterizations could be given in description logics, and finally apply the results to mixtures.

**Results:**

At first sight, collectives and compounds seem to be disjoint categories. Their disjointness, however, relies on agreement about what are uniform entities, and thus on the granularity of description. For instance, the distinction between isomeric subtypes of a molecule can be important in one use case but might be neglected in another one. We demonstrate that, as implemented in the BioTop domain upper level ontology, equivalence or subsumption between different descriptions of same or similar entities cannot be achieved. Using OWL-DL, we propose a new design pattern that avoids primitive subrelations at the expense of more complex descriptions and thus supports the needed inferences.

## Background

### Mereology in the biomedical domain

In biomedical ontologies, mereological relations between parts and wholes [1] have always been conferred a special importance due to their relevance for describing the structural makeup of material entities such as body parts, cells, cell components, and biomolecules ([1-3]). Numerous subrelations of the foundational **has_part** relation, relevant for the biomedical domain, have been proposed in the context of biomedical ontologies such as GALEN [4] or the Foundational Model of Anatomy (FMA) [5]. In BioTop, a top-domain ontology for the biomedical domain ([3,6,8]), the number of relations has been restricted to a minimum, mostly following the precepts of the OBO relation ontology [7]. However, the need for two distinct mereological relations, **has_grain** and **has_component**, both subrelations of **has_proper_part**, has been advocated. BioTop builds upon the formal design principles propagated by the OBO Foundry initiative ([10,11]) and is implemented in OWL [12], the standard ontology language of the Semantic Web, using its description logics specification OWL DL. We will first present the approach of BioTop, discuss it critically, and then present a new suggestion and discuss whether it can and should be applied to mixtures, too.

### The BioTop approach

Inspired by the work of Rector et al. [13], Schulz et al. adduce various criteria to distinguish between grains and components [2]: Grains are the constituting elements of homogeneous collections, such as the sheep in a flock or the H_2_O molecules in a drop of water. Components are the constituting elements of a compound constituted by well identified parts, such as a bicycle being composed of frame, wheels, saddle, front set etc, or a skull composed of neatly distinguishable bones.

In BioTop’s account of compounds and components, it is necessary for a compound that its sortal identity depends on the exact sum of its components. In contrast, the sortal identity of a collective does not. The example given is a portion of water, from which a drop is removed: What is left behind is still a portion of water, i.e. an entity of the same type. Removing a nucleotide from a sequence of nucleotides, on the other hand, brings into existence an instance of a structurally different type of sequence. Secondly, BioTop claims that grains unlike components are not spatially connected – a criterion which is not further expanded on for lack of an uncontroversial model of connection.

For their formal characterization, however, they use still other properties of the relations: In the relation **has_component**, no two components of a compound are overlapping, and the components exhaust the whole compound. In the relation **has_grain** all grains must be of the same type and any two grains are spatially disconnected, while all grains together exhaust the whole (see Table 1).

**Table 1:** Defining properties of collections and compounds in BioTop

Collections	Compounds
- Grains belong to one kind only.	- Components can belong to diverse kinds.
- Grains are spatially disconnected	- Components have no proper overlap.
- Number of grains is not essential.	- Number of components is essential.

### Problems

Schulz et al. [2] present their formal characterization as giving both necessary and sufficient conditions for the respective relations, i.e. as definitions. However, their formal descriptions are not unproblematic. It is lost in the formalization that any complex thing can be partitioned in various ways, and any component is a component only with respect to a certain partition of the compound.

Many of these formal shortcomings can easily be repaired. Not as easily resolved, however, is the problem that these formal characterizations do not at all capture the criteria to draw a clear distinction between grains and compounds. Rather, as the definitions stand, all grains are also components. For having no spatial overlap is a necessary condition for being spatially disconnected, and thus everything that is spatially disconnected has no spatial overlap. As can be seen from the criteria discussed in the preceding section (cf. Table 1), this result is not at all intended.

Moreover, the two categories of BioTop are not sufficient to deal with all types of complex entities. This can be seen when we look at the distinguishing features of these categories: Collectives are spatially unconnected, mono-sortal and flexible with regard to the number of their grains, whereas compounds are spatially connected, multi-sortal and strict with regard to the number of their components. Thus both collectives and compounds are characterized by three features that are, in fact, logically independent from each other and can thus vary independently. E.g., there could be complex entities just like compounds without a spatial connection of their components, and there could be connections other than spatial contiguity. Other compounds could show some flexibility with regard to the numbers of their members. All possible combinations can be seen in Table 2. Of all the fields in the table, both BioTop and Rector et al. [13] up to now provide for two only: They provide only for what we will call “flexible collectives” and “strict connected compounds”. It does neither provide for “strict collectives” nor “flexible compounds”, both of which will be discussed in this paper (cf. Table 3 for examples). It is thus necessary to take a fresh look at collectives and compounds.

**Table 2:** Types of complex entities in BioTop and Rector et al.

	unconnected; mono-sortal	connected; possibly multi-sortal
**flexible** **re. number**	BioTop: collectives Rector et al.: granular parthood	

**strict** **re. number**		BioTop: compounds Rector et al.: determinate parthood

**Table 3:** Types of complex entities discussed in this paper

	mono-sortal	multi-sortal
**flexible** **re. number**	“flexible collectives” (e.g., a portion of water)	“flexible compounds” (e.g., a hand)

**strict** **re. number**	“strict collectives” (e.g., a pair of kidneys)	“strict compounds” (e.g., a propanol molecule)

## Results

### Flexible collectives and their grains

Grains are constituent parts of pluralities: Herds are pluralities of cows, bacteria colonies are pluralities of bacteria, and water samples are pluralities of water molecules. Such pluralities of grains are often called collectives. Grains and collectives are closely related: Grains are grains of collectives and collectives are collectives of grains. To be a collective and to have grains are thus two sides of the same coin.

In order to capture this idea we use a COLL-index as an operator that takes an arbitrary type and yields a type of collectives of instances of the original type [6]. Instances of a collective type *X_COLL_* are collectives whose grains are instances of the type *X.* It is no easy question how many grains you need for a heap and how many cows you need for a herd. As the COLL-index needs to provide for many diverse cases, we cannot set any non-trivial minimum requirements. To make things easier (and consistent with earlier work [14]), we allow for collectives with one grain only. Thus not every cow-collectives in the sense defined by us is a herd, and not any grain-collective is a heap. However, we go not as far as Rector et al., who allow even for collectives with no grains at all ([13], p. 338). Were we to allow such empty collectives, every empty collective would be an instance of any collective type. We thus postulate as axioms for the COLL-index:

(Grain-Existence) *x*** instance_of*** X_COLL_* ↔ Ǝ*y* (*y* instance_of *X* ⋀ *y*** grain_of*** x*)    (1)

(Mono-Sortality) *x*** instance_of*** X_COLL_* ↔ ∀*g* (*g*** grain_of*** x* ⊃ *g*** instance_of*** X*)    (2)

With the help of the COLL-index and the **instance_of** relation we can now state what it is to be a grain:

*x*** has_grain*** y* ⇔_def_ (*x*** has_proper_part*** y*) ⋀           (3)

  Ǝ*X* Ǝ*Y* (*x*** instance_of*** X* ⋀ *y*** instance_of*** Y* ⋀ *X is_a Y_COLL_*)

The first conjunct guarantees that **has_grain** is a sub-relation of **has_proper_part** (and thus also a sub-relation of **has_part**). The last conjunct guarantees both that all grains in question are grains of the same type (i.e. of type *Y*) and that there are no grains of *x* that are not *Y*s (i.e. that the *Y*s exhaust the whole *x*). Rector et al. postulate that the **has_grain** relation is irreflexive, anti-symmetric, non-transitive, and propagates via the **is_part_of** relation. These properties follow as corollaries from our definition. Table 4 presents a list of these theorems with proofs.

**Table 4:** Properties of the **has_grain** relation (in first order logic)

Theorem	Proof
*Irreflexivity* ∀*x* ~(*x*** has_grain*** x*)	Assume that *x*** has_grain*** x.* But then, by definition, *x*** has_proper_part*** x*. This is impossible, thus the proposition follows.

*Symmetry* ∀*x* ∀*y* (*x*** has_grain*** y* ↔ ~(*y*** has_grain*** x*))	Assume both that *x*** has_grain*** y* and that *y*** has_grain*** x.* But then, by definition, *x*** has_proper_part*** y*, as well as *y*** has_proper_part*** x*. This is impossible, thus the proposition follows.

*Non-Transitivity* ~∀*x* ∀*y* ∀*z* (*x*** has_grain*** y* ⋀ *y*** has_grain*** z* ⊃ *x*** has_grain*** z*)	Proof by non-transitive example: A galaxy is a star collection and a star is a molecule collection, but a galaxy is not a molecule collection.

*Propagation I* ∀*x* ∀*y* ∀*z* (*x*** has_part*** y* ⋀ *y*** has_grain *z*** ⊃ *x*** has_part*** z*)	Follows from the transitivity of **has_part** and the fact that** has_grain** implies **has_proper_part**.

*Propagation II* ∀*x* ∀*y* ∀*z* (*x*** has_grain*** y* ⋀ *y*** has_part*****z*** ⊃ *x*** has_part*** z*)	Follows from the transitivity of **has_part** and the fact that **has_grain** implies **has_proper_part**.

It should be noted that we deviate from the former BioTop category in that we do not add any requirement concerning the disconnectedness of grains. Hence it does not matter whether the grains of a collective happen to be connected or disconnected. A collection of water molecules may be disconnected when existing in gas state, while having connections in various degrees when in liquid or solid state. In which state whatsoever it exists, whether the grains are connected or not, it is still a collection of water molecules.

### Strict compounds and their components

A component is always a component of a compound with respect to a certain partition of this compound. A protein chain, for example, can be partitioned into its constituent amino acid monomers, but it can also be portioned into the atoms it consists of. These cases differ with respect to their level of granularity, but a partition may also arbitrarily crisscross granularity levels, e.g. by portioning half of a protein chain into monomers and the other half into atoms. Like many terms ending with “-ion”, “partition” features a product-process-ambiguity. “Partition” can denote the (mechanical or cognitive) act or process of dividing something into parts. We will, however, use the term to denote the product of such a process, i.e. a collection of parts that make up the whole. These parts can be called the “segments” of a partition. The segments of a partition are pairwise disjoint and jointly exhaust the whole compound. That is:

• If *p_1_* and *p_2_* are two distinct segments of the same partition *P*, *p_1_* and *p_2_* have no spatial overlap.

• The mereological sum of all the segments of a partition overlaps completely the compound, and *vice versa*.

The segments of a partition form a collective entity that is an independent continuant. We can describe a partition by enumerating its segments.

Not all segments are components, because not all partitions are partitions of a compound into components. Let *p* be a partition consisting of the segments *p_1_*, *p_2_*, .., *p_n_* (with n > 1). If all segments are instances of the same type of independent continuants, the partition can be regarded as establishing a collective consisting out of *n* grains. According to the intuitive specification described above (cf. Table 1), it is not necessary for a compound that all components are of the same type, but their number is essential. We can thus define a strict compound as follows:

• Let *p* be a partition consisting of the segments *p_1_*, *p_2_*, …, *p_n_*. Then *p* is a strict compound of type *X* if and only if it is not possible to add further segments *p_n_*_+_*_1_*, *p_n_*_+_*_2_*, … or to subtract any of the segments in such a way that the resulting sum is still an instance of *X*.

We can now proceed to define the relation **has_component**. More specifically, as we have defined a compound with reference to a certain partition, we have to start with the component-relation relativized to a certain partition:

• *x*** has_component*** y*** with_respect_to_a_partition*** p* if and only if the partition p of x is a compound consisting of *p_1_*, *p_2_*, …, *p_n_* and *y* = *p_i_* for some 1 ≤ *i* ≤ *n*.

It is only now that we can define the unrelativized component-relation:

• *x*** has_component*** y* if and only if there is some segment *p_i_* of some partition *p* of *x*, such that *y* = *p_i_*.

As the relation **has_component** makes no reference to any specific partition, this relation might in many cases be too weak and thus uninformative. All that remains is the requirement that *y* has to be a part of *x*, or rather (as we set *n* > 1 in the definition of a compound) a proper part. If we require that the partition in question is on a certain level of granularity *G* (with *G* indicating the partition level by being a placeholder for, e.g. *Molecule* or *Atom*), we can restrict the granularity of the components to the required level. We can thus express

• “being a molecular component of *y*” as:

*x*** proper_part_of*** y* ⋀ *x*** instance_of*** Molecule*   (4)

• “being an atomic component of *y*” as:

*x*** proper_part_of*** y* ⋀ *x*** instance_of*** Atom*   (5)

Alternatively, we can set up a complete list of all possible types of components to be found on a certain type of partition. If we want to partition a thing into the subatomic particles contained in the standard model of the atom, we divide it in protons, neutrons and electrons. We can thus express “being an atomic particle component of y” as

*x*** proper_part_of*** y* ⋀         (6)

  (*x*** instance_of*** Proton* ∨ *x*** instance_of*** Neutron* ∨ *x*** instance_of*** Electron*)

We do not have to add any further requirement to ensure that components have no proper overlap, because this is already entailed by the logical properties of partitions. In not adding further requirements to ensure that components are spatially connected, we deviate from BioTop. According to the definition given above, the components of a compound may or may not be spatially connected to each other. The components of the skull are connected to each other, while the components of a chamber music quartet are normally spatially disconnected. Nevertheless, the chamber music quartet is a full blown compound: Under the canonical partition, it has four components, the subtraction of one of which would put an end to the quartet (for then it would be a trio). The existence of skulls or molecules, however, requires that the components are in fact spatially connected to each other: Two hydrogen atoms and an oxygen atom only form a water molecule if they are bond together, and a number of bones does not form a skull if they are scattered in, say, several boxes. This, however, has to be ensured with the help of additional mereotopological means (cf., e.g., [15,16]).

### Varieties of compounds

The account of compounds that we presented in the preceding section has two serious shortcomings: On the one hand, as we have already explained, it is too strict to deal with flexible compounds. On the other hand it is blind to the number of components of the same kind and it is blind to structure. That both elements are important for a complete account of compounds can easily be seen when we consider words as compounds of letters: The words “man” and “manna” contain only tokens of the same type of letters, but they contain them in different numbers. But so far our account of compounds cannot distinguish between these words. Moreover, the words “three” and “ether” are clearly distinct, although they share the same letters, and although these letters occur in the same numbers in both words. But they are distinguished by their structure, i.e. in this case by the serial order of the letters in the word.

Similar examples can be derived from chemistry: Hydrogen peroxide (H_2_O_2_) contains the same kind of atoms as do water molecules (H_2_O), but they are not the same kind of molecule. And the various isomers of, say, propanol (both with the sum formula C_3_H_8_O), share both the atom types and the number of atom tokens, but are distinguished by their structure.

The problem of structure blindness, on the one hand, can only be solved with the help of additional means, e.g. through the application of mereotopological relations (cf. again [15,16]); this, however, lies outside of the scope of the present paper. The problem of number blindness, on the other hand, can be solved through combining our account of compounds with our account of collectives.

### Strict collections

Starting from our definition of collectives, we can define subtypes of collectives that have a certain cardinality, i.e. that consist of a certain number of grains. We can, for example, define a PAIR-operator as follows:

*x*** instance_of*** X_PAIR_* ⇔_def_             (7)

Ǝ*y* Ǝ*z* (*x*** has_grain*** y* ⋀ *x*** has_grain*** z* ⋀ *y*** instance_of*** X* ⋀ *z*** instance_of*** X*

⋀ *x* ≠ *y* ≠ *z* ⋀ ∀*u* (*x*** has_grain*** u* ⊃ (*u* = *y* ∨ *u* = *z*))

Along this line, we can also define trios, quartets, quintets, and, in general, strict collections of any cardinality. We will call a strict collective with *n* grains an n-collective, and we will use the subscript “*n-COLL*” as an operator yielding such a *n-*collective. In DL, we can use the already built-in modifier “exactly *n*” to model such strict collectives with *n* grains ([17], p. 529).

### Solution for number blindness

With the help of strict collections we can now better define strict compounds and fix, e.g., the number of atoms of an H_2_O molecule and thus distinguish it from, say, a hydrogen peroxide:

*x*** instance_of*** Water molecule* ⊃               (8)

Ǝ*y* Ǝ*z x*** has_component*** y* ⋀ *y*** instance_of*** O-Atom* ⋀ ∀*g* ((*x*** has_component*** g* ⋀

*g* instance_of *O-Atom*) ⊃ *g* = y) ⋀ *x*** has_component*** z* ⋀ *z*** instance_of*** H_PAIR_* ⋀

∀*g* ((*x*** has_component*** g* ⋀ *g*** instance_of*** H-Atom*) ⊃ *g* grain_of *z*)

Thus our strategy is to heal number blindness by defining these compounds as compounds of strict collectives.

### Flexible compounds

Though our definition of strict compounds fits the specification given by Schulz et al. [2], it might be too strong a requirement for certain purposes. A canonical hand of a human being, for example, is a strict compound of five fingers (plus other components). But it is possible that a hand is not well-formed, e.g., when someone has lost a finger or when a baby is born with six fingers at its hand. The type *Hand* is flexible enough to cover all these cases, because it is a supertype both of *Canonical_Hand* and of *Non_canonical_Hand*. Also, while the number of carbon atoms is essential for an ethanol molecule, it is no longer essential when we describe the molecule less specifically as an alcohol molecule, which could have any number of carbon atoms. Let us call these cases “flexible compounds”. Again we can model flexible compounds by combining our account of compounds with our account of collectives. In a nutshell, flexible compounds are compounds of flexible collectives. I.e, for the examples of the hand and of the arbitrarily long C-backbone of an alcohol molecule:

∀x (x **instance_of*** Hand* ⊃ Ǝy (*x*** has_component*** y* ⋀ *y*** instance_of*** Finger_COLL_*)       (9)

∀x (*x*** instance_of*** Alcohol-molecule* ⊃             (10)

  Ǝy (*x*** has_component*** y* ⋀ *y*** instance_of*** C_COLL_*)

These two cases are, of course, of a quite different nature, as the alcohol molecule gains its flexibility only through the unspecific description. When we describe a given token of an alcohol molecule as, say, an ethanol molecule, it is not be flexible at all with respect to the number of its C-atoms. This shows that flexibility sometimes comes with less specificity.

We note in passing that according to our definition of the COLL-modifier, the formula (9) given above implies that there is no hand without any finger. That seems to be objectionable. This problem does not arise in the alcohol example, for even methanol molecules have one carbon atom.

### Putting it into description logics

Our definition of the relations **has_grain** and **has_component** has required first order logics. Automated reasoning over non-trivial ontologies, however, needs a computational subset of first order logic. We use Description Logics (DL) together with the HermiT reasoner [18], using Manchester syntax [19] as a user-friendly compact syntax for OWL 2 ontologies. In the rest of this paper we will study how this reduced logic is capable of representing our notions of grain- and componenthood. For a translation of the above formulae into OWL see Table 5. We will here take a closer look at formula (8). We can render (8) into description logic as follows:

**Table 5:** Translation of first order statements into DL

No.	Translation into DL	Remark
(4)	*Molecule* and **proper_part_of** some *X*	“being a molecular component of *X*”

(5)	*Atom* and **proper_part_of** some *X*	“being an atomic component of *X*”

(6)	(*Proton* or *Neutron* or *Electron*) and **proper_part_of** some *X*	“being an atomic part of *X*”

(7)	*X_COLL_* and **has_grain** exactly 2 *X* and **has_grain** only *X*	“being an *X_PAIR_*”

(9)	*Hand* subClassOf **has_component** some *Finger_COLL_*	

(10)	*Alcohol-molecule* subClassOf **has_component** some *C_COLL_*	

*Water molecule* subClassOf             (11)

  **has_component** exactly 1 *O-Atom* and

  **has_component** exactly 1 (*H-Atom_COLL_* and **has_grain** exactly 2 *H-Atom*)

We can define *H-Atom_COLL_*, in turn, as:

*H-Atom_COLL_* equivalentTo             (12)

  **has_grain** some *H-Atom* and **has_grain** only *H-Atom*

Slightly less clumsily, we could replace (11) plus (12), with (13):

*Water molecule* subClassOf             (13)

  **has_component** exactly 1 *O-Atom* and

  **has_component** exactly 2 *H-Atom* and

  **has_component** only (*H-Atom* or *O-Atom*)

Independently of which of these two versions we choose, however, we encounter the problem that the number of grains is not transparent to existing reasoners. Both with (11) + (12) and with the alternative (13), a query for all molecules that have exactly three atoms as part or as components, i.e.

  **has_proper_part** exactly 3 *Atom*             (14)

  **has_component** exactly 3 *Atom*             (15)

does not return the class *Water molecule*, due to the fact that current reasoners do not allow to use transitive relations in number restrictions in order to preserve decidability [20].

This is also the reason why we cannot recommend the following solution, forgoing the use of the special relation **has_component** and using the general **has_proper_part** only:

*Water molecule* subClassOf             (16)

  **has_proper_part** exactly 1 *O-Atom* and

  **has_proper_part** exactly 2 *H-Atom* and

  **has_proper_part** only (*H-Atom* or *O-Atom* or not *Atom*)

Because of its transitivity, the general relation **has_proper_part** can therefore only be used where number restriction are not necessary. This is, in effect, the strategy we will further develop in the next section for the formal representation of mixtures.

### Representing mixtures

We will now apply the notions of grains and components to the area of biomedicine and biochemistry. In biological systems, we practically never encounter pure substances such as 100 % alcohol or pure oxygen. Whether we deal with body substances, with tissues, or with cell protoplasm, the normal case is a mixture of grains of different kinds and sizes. A complete enumeration of all those different kinds is often neither possible nor desirable. There are at least three different ways to represent this situation using grains and components. We will describe these ways using the conventional standard, according to which formal relations are rendered with an all-some semantics.

First, we could dispose of the uniformity condition for the **has_grain** relation and allow that a collective may have grains of different sorts, e.g.:

*BloodPlasmaSample* subclassOf

  **has_grain** some (*AlbuminMolecule or GlobulinMolecule*)     (17)

This, however, is a severe modification of the underlying logical structure that weakens the **has_grain** relation and puts at risk the expressive power gained through the introduction of that relationin the first place.

Second, we could refrain from a sortal distinction between the grains and subsume them under their most specific common supertype, e.g.:

*BloodPlasmaSample* subclassOf     (18)

  **has_grain** some *PlasmaProteinMolecule*

Third, we can define mixtures as *compounds of fractions*, each fraction in turn being a collective consisting out of grains of the same sort:

*BloodPlasmaSample* subclassOf     (19)

  has_component some

    (**has_grain some*** AlbuminMolecule*) and

    (**has_grain some*** GlobulinMolecule*)

With option 1 we would sacrifice the ontological purity of the proposed notion of grainhood, although its simplicity would offer some advantages for ontology engineers. Option 2 would need backing by a representation of which molecules are in fact plasma protein molecules, and it would lead to extreme compromises such as to regard all plasma  protein molecules as entities of the same kind when describing blood. Option 3 would be the one that is most consistent with the approach proposed in this paper, as it neatly distinguishes compounds from collectives. Formally representing mixtures as compounds of fractions is cognitively adequate to the ways biologists and chemists think. For instance, the concept of substance concentration hinges on a view of the proportion of different substance fractions.

**Figure 1:**
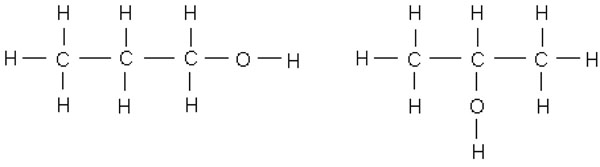
Propanol isomers: N-Propanol (left), I-Propanol (right)

Option 3, however, is prone to produce interoperability problems. In order to demonstrate this, we will discuss the use case of propanol, which is a common disinfectant. The sample ontology is available in the Additional File 1 (propanol.owl) belonging to this paper. Propanol is a type of organic molecule with the two isomers *N-Propanol* and *I-Propanol* (Fig. 1).

One way to describe a collection of propanol, regardless which isomer it contains, is the following:

*Propanol_COLL_* equivalentTo     (20)

  (**has_grain** some *Propanol_Molecule*) and

  (**has_grain** only *Propanol_Molecule*)

Accordingly,

*I-Propanol_COLL_* equivalentTo     (21)

  (**has_grain** some *I-Propanol_Molecule*) and

  (**has_grain** only I-Propanol_Molecule)

*N-Propanol_COLL_* equivalentTo     (22)

  (**has_grain** some *N-Propanol_Molecule*) and

  (**has_grain** only N-Propanol_Molecule)

A mixture of *I-Propanol* with *N-Propanol*, i.e. a compound of two fractions which are collectives is then represented as follows:

*Propanol_Mixture* equivalentTo     (23)

  (**has_component** some *N-Propanol_COLL_*) and

  (**has_component** some *I-Propanol_COLL_*) and

  (**has_component** only (*Propanol_COLL_* or not *Molecule*))

The first two conjuncts assure that the mixture contains at least one molecule of n-propanol and at least one molecule of i-propanol. The third conjunct guarantees that the only molecules in the mixtures are propanol molecules.

Propanol mixtures should be classified as Propanol collections, because they contain only propanol molecules. In order to achieve interoperability between descriptions of different specificity levels, it would therefore be desirable that the subsumption of *Propanol_Mixture* by *Propanol_COLL_* be computed by logical reasoning. This can, however, not directly be implemented with the available description logics, because the criteria that distinguish **has_grain** and **has_component** from its superrelation **has_proper_part** are not expressible in description logics. A practical solution which supports the desired inferences has to refrain from the use of these subrelations of **has_proper_part**. It is, however, possible to define *Propanol_Mixture* without these relations:

*Propanol_Mixture* equivalentTo     (24)

  (**has_proper_part** some N-Propanol_Molecule) and

  (**has_proper_part** some I-Propanol_Molecule) and

  (**has_proper_part** only (*Propanol_COLL_* or

   **proper_part_of** some *Propanol_COLL_*))

Collectives can be defined analogously along the following line:

*Propanol_COLL_* equivalentTo     (25)

  (**has_proper_part** some *Propanol_Molecule*) and

  (**has_proper_part** only

   (Propanol_Molecule or not (Molecule)))

Once mixtures and collectives are defined in this way, it trivially follows that all propanol mixtures are propanol collectives, too. A description logics classifier can automatically compute a subclass relation between *Propanol_Mixture* and *Propanol_COLL_*, and we thus assure cross-granular interoperability.

## Discussion

### Granularity and specificity

Our presentation above already indicates that mixtures and collectives are not distinct categories. This is because the properties of mono-sortality and multi-sortality are both granularity dependent and specificity dependent. A collective of fruits in a basket can at the same time be a mixture of apples and pears: Here, mono-sortality is lost with a more specific description of the grains. Mono-sortality can also be lost with a re-description on a lower granular level: What is a mono-sortal collective on the molecular level, like, e.g., a collective of water molecules, is a multi-sortal on the atomic level. Sometimes mono-sortality can also be gained with a re-description on a lower granular level: When seen on the atomic level, a mixture of oxygen molecules and ozone molecules turns out to have grains of the same sort only, i.e. oxygen atoms.

For this reason, our design patterns forgo a strict dichotomy of mono-sortal and multi-sortal complexes. Instead, we use the neutral part-of relation, together with granularity-indicating criteria. This allows us, to a degree, reasoning across granular levels.

### COLL-Index

Though we have successfully put to work the new design patterns, there are still several issues that have to be dealt with. Up to now, we have not thoroughly formalized the COLL-Index, and even if we did, current ontology editors and reasoners were not able to process it. Thus the only thing we can recommend at the present stage is to use it as a naming convention, especially for the use of human ontology developers. Any attempt to formalize the COLL-Index will have to deal with such issues as whether, e.g., *Entity_COLL_* is itself a subclass of *Entity*.

### Connectedness

Up to now, our whole approach is agnostic to connectedness. An effect of this is, e.g., that we cannot distinguish between bunches of oxygen atoms, oxygen molecules or ozone molecules (i.e., in sum formulae, between collectives of O, O_2_, and O_3_).

### Collectives of other top-level categories

In this paper, we have only described collectives of independent continuants. For the biomedical domain, however, it is also very useful to consider collectives of occurrents like processes [14], and we could also think about collectives of dependent continuants. Also, collectives of entities belonging to different top-level categories could be taken into account. An interesting approach could be, e.g., to model a molecule as a compound not only of atoms, but as a compound of atoms and chemical bonds. This approach would consider the connections, hitherto neglected in this paper, as components of compounds in their own right.

## Conclusion

Previous work had suggested that collectives and compounds are disjoint categories that together exhaust the realm of complex entities. We have, however, shown that these two categories are only the extreme opposites in a wide spectrum of complex entities, and that they are far from exhaustive. Their supposed disjointness, however, relies on agreement about what are uniform entities, and thus on the specificity of description. While the **has_proper_part** subrelations **has_grain** and **has_component** can be used to characterize biomedical entities like mixtures, description logic reasoners fail to calculate equivalence or subsumption relations between different descriptions of the same or similar entity classes. We demonstrated that using a different ontology design pattern that avoids primitive subrelations at the expense of more complex descriptions supports the needed inferences using description logics. Our example also provides evidence that compounds and collectives are no disjoint categories. The reason for this is that the assertion of sameness depends on the specificity or level of detail with which the members are described. A collection may appear mono-sortal under a view which ignores minor differences, such as configuration of isomers or the different number of neutrons in isotopes. The same thing in reality, however, may become multi-sortal under a perspective which regards these variations as differentiae for further subclassification.

Although the distinction between grains and components seems dispensable for the use cases for which description logics representations are adequate, relations such as **has_grain** and **has_component** are nevertheless useful for precise ontological descriptions, e.g. through providing background knowledge for the human modeler. Furthermore they may be needed for axioms that state the exact number of parts, as the combination of transitive relations (such as **has_proper_part**) with number restrictions is not supported by DL reasoners. This requires, on the one hand, a strict axiomatization in order to prevent modeling errors on the part of human modelers, although axioms in first order predicate logic cannot be used for tractable reasoning. On the other hand it must be assured that the desired (and needed) inferences are not blocked. This goal can be attained by well-defined simplification patterns. It must be emphasized, however, that reasoners do not cope with cardinalities of parts; therefore support for inferences including cardinality cannot be expected.

## Competing interests

The authors declare that they have no competing interests.

## Authors' contributions

The authors contributed equally to this work. LJ focused on the axiomatization of the relations in first order logic; SS focused on the simplified description logic implementation.

## Supplementary Material

Additional file 1 **Ontology example** This example demonstrates OWL-DL definitions of mixtures and collectives according to Formula (24) and (25), using the basic mereological relations has_proper_part and proper_part_of, but not has_grain and has_component. Three variants of mixture representations are distinguished; all of them are classified as collectives. Click here for file
